# Influence of mobility restrictions on post‐stroke pain

**DOI:** 10.1002/brb3.2092

**Published:** 2021-03-02

**Authors:** Pelin Atalan, Guna Bērziņa, Katharina S Sunnerhagen

**Affiliations:** ^1^ Institute of Health Sciences Department of Physiotherapy and Rehabilitation Gazi University Ankara Turkey; ^2^ Department of Rehabilitation Riga Stradiņš University Riga Latvia; ^3^ Institute of Neuroscience and Physiology Section for Clinical Neuroscience The Sahlgrenska Academy University of Gothenburg Göteborg Sweden

**Keywords:** Comorbidity, Mobility, Pain, Stroke, Vitality

## Abstract

**Objectives:**

The objective of this study was to investigate the role of mobility limitations and vitality, as well as additional factors such as comorbidities, to predict post‐stroke pain.

**Materials & Methods:**

This study included cross‐sectional data from 214 participants living in varied settings in different parts of Sweden. Participants were asked to complete the Stroke Impact Scale, Medical Outcomes Study Short Form 36, and Self‐administered Comorbidity Questionnaire to evaluate mobility, vitality, comorbidities, and pain. Descriptive statistics were used for demographic and clinical characteristics. Binary logistic regression analysis was performed to predict the pain domain score on Medical Outcomes Study Short Form 36.

**Results:**

The mean age of all participants in the sample was 66 years (*SD* 14); 43.4% of the study population were women. After analyses, “standing without losing balance and vitality’’ were found to be significant predictors in the model which explained the pain score on Medical Outcomes Study Short Form 36.

**Conclusions:**

In conclusion, the results suggest that restrictions in mobility and low vitality have an important role on the occurrence of post‐stroke pain. Having post‐stroke pain could be due to not able to stand without losing balance and low vitality. Thus, rehabilitation professionals may consider the importance of these factors, especially mobility restrictions, in preventing post‐stroke pain.

## Graphical abstract

The objective of this study was to investigate the role of mobility, limitations, and vitality, as well as additional factors such as comorbidities, to predict post‐stroke pain. The results suggest that restrictions in mobility and low vitality are important factors for the occurrence of post‐stroke pain.

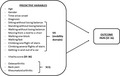

## 1 INTRODUCTION

Stroke affects 1 in 4 people worldwide (Collaborators GLRoS, 2018) and is one of the most common causes of death around the world with a variable rate of mortality (2.2‐298/100.000/year) (Thrift et al., 2017). The neurological consequences of stroke can affect many aspects of patients’ lives (Schöttke & Giabbiconi, 2015; Sun et al., 2014). Stroke survivors report a variety of stroke‐related outcomes, including pain, disability, physical inactivity, cognitive impairment, anxiety, and depression, even 10–15 years after the first incidence of stroke (Crichton et al., 2016).

Post‐stroke pain (PSP) is one of the common long‐term stroke‐related outcomes, with varied prevalence (10%–45.8%) (Jonsson et al., 2006; Kim, 2009), and may occur in different parts of the body at different intensities. The prevalence of PSP differs between the acute, subacute, and chronic phases post‐stroke, with the highest prevalence of PSP in the subacute phase (Paolucci et al., 2015). As we all accept, pain is a discomfort feeling, and it has negative effects on varied physical, social, and emotional functions for both stroke patients and healthy people (Galligan et al., 2016; Jönsson et al., 2006; Miller et al., 2013; Stroemel‐Scheder et al., 2020). In a review, it is been highlighted that PSP is leading to anxiety, depression, and low physical function as well as quality of life (Payton & Soundy, 2020). In clinical practice, progress in rehabilitation sessions becomes difficult for some patients because of PSP, which is generally under‐recognized (Harrison & Field, 2015) and often neglected (Henon, 2006). While the specific mechanism(s) behind PSP are not well understood, some have been elucidated. These include musculoskeletal problems and neurological damage, such as spasticity (Wissel et al., 2010), upper extremity weakness (Gamble et al., 2002), stroke severity, (Appelros, 2006) and sensory deficits (Sommerfeld & Welmer, 2012). It has been also shown that PSP is correlated with cognitive decline, fatigue, depression, and lower quality of life (Harrison & Field, 2015; Westerlind et al., 2020). As rehabilitation practice aims to improve functionality and overcome factors that may restrict patients’ daily activities (Dobkin, 2004), it is important to understand the predictors of PSP that may limit both functionality and daily activities (Naess et al., 2012).

Mobility, which simply means someone's ability to move, is one of the key aims of rehabilitation in stroke patients. The mobility of stroke survivors is highly affected due to motor and sensory deficits. Slow walking, (Schmid et al., 2007) impaired balance, (Said et al., 2008) increased risk of falls, (Mackintosh et al., 2005) or not being able to sit, stand, or walk are some of the factors leading to mobility limitations. A very common question that patients and their caregivers ask health professionals is whether they will walk again. Considering that patients want to be mobile and walk as soon as possible, it is understandable that restricted mobility can affect stroke patients both physically and mentally. It is known that physical function is one of the restricted things due to pain (Hart et al., 2000). It is also shown that stroke patients who were restricted in mobility were more likely to experience more frequent pain even after 5 years after stroke onset (Westerlind et al., 2020). However, it has not been investigated patients’ mobility levels, how much difficulty they experience when they are performing different mobility tasks, and effects of those on post‐stroke pain.

Vitality is defined as one's conscious experience of possessing energy and aliveness (Ryan & Frederick, 1997). Vitality is necessary for building social interactions and functions (Kawachi & Berkman, 2001). We presume that a socially and physically constrained stroke patient, due to mobility restrictions and low vitality, may feel pain more than others without such restrictions. Especially, neurologically affected patients feel more depressed due to physical inactivity (Miller et al., 2013). Also, it has been determined that post‐stroke depression and functional dependence are highly related to each other (Brown et al., 2012). Therefore, we hypothesized that mobility restrictions and low vitality may be some of the possible factors responsible for PSP.

Considering all possible factors, the aim of this study was to determine the role of different mobility restriction levels and vitality, as well as additional factors such as comorbidities and demographic details in predicting post‐stroke pain (PSP).

## 2 MATERIALS AND METHODS

### 2.1 Participants

The convenience sample included 214 participants living in varying types of communities (city, small communities, countryside, and sparsely populated areas) in different parts of Sweden. The patients had been in contact with a rehabilitation medicine unit, a primary care physical therapist, or were recruited through a local branch of the Swedish Stroke Association. The inclusion criteria were diagnosis of stroke (ICD‐10 codes 160–167) as assessed by a specialist according to WHO criteria, confirmation by computed tomography, and they were at least 3 months from stroke onset. In addition, all participants were at least 18 years old, and they or their next‐of‐kin gave informed consent before the evaluations.

Participants were asked to complete the Stroke Impact Scale (SIS), Medical Outcomes Study Short Form 36 (SF‐36), and Self‐administered Comorbidity Questionnaire (SCQ) in their home, an outpatient clinic, or during a visit to the university department, with assistance from health professionals (doctors, nurses, occupational therapists, and physiotherapists) who were experienced in stroke rehabilitation.

The study conformed to the ethical principles of the Declaration of Helsinki and was approved by the ethics committee of the University of Gothenburg (numbers T129‐05/Ad 419–04 and 390–05). All patients were provided oral and written information on the study and provided written informed consent for participation.

## 3 DATA COLLECTION

### 3.1 Stroke Impact Scale (SIS)

The SIS estimates the effects of stroke on an individual's health and life (Duncan et al., 1999). The SIS consists of 59 different items, which are divided into related stroke outcomes and an extra question regarding how much the patient feels recovered after stroke. The nine items about mobility were used in this study as predictive variables. Each item is rated from 1 to 5, with 1 representing “having trouble all of the time” and 5 representing “having trouble none of the time”). The answers to the questions were dichotomized; a response of 1–4 was re‐coded as 1 (difficulty) and 5 was re‐coded as 0 (no difficulty) to better understand and use in demographics how frequent the difficulties in mobility items.

### 3.2 Medical Outcomes Study Short Form 36 (SF‐36)

The SF‐36 is a commonly used comprehensive, patient‐reported measure designed to assess health‐related quality of life (Haan, 2002). The SF‐36 is divided into eight different domains (physical functioning, role limitations due to physical problems, general health perceptions/physical component, social functioning, general mental health, role limitations due to emotional problems, pain, and vitality/mental component) and has two questions comparing health status a year ago and now. In the present study, the “vitality” domain was used as one of the predictive variables and the “pain” domain as the outcome. An average score (0–100) for the overall questions, for each domain and for every single question, can be calculated out of 100 points separately, and the average score of the four items in the vitality domain was scored 0 (low vitality) to 100 (high vitality). The answers to the question regarding how much bodily pain patient feel were dichotomized, a response of 2–6 was re‐coded as 1 (pain) and 1 was re‐coded as 0 (no pain).

### 3.3 Self‐administered Comorbidity Questionnaire (SCQ)

The SCQ (Swedish version) is an effective measurement for assessing co‐morbid conditions (Sangha et al., 2003). The SCQ includes 13 common problems and allows an indication of additional unlisted medical problems. Three questions apply to each problem: whether the patient has the health problem, whether the patient is receiving treatment for that problem, and whether the problem limits their activities. Each question can be answered “yes” or “no”; in this study, missing answers were also treated as “no.” The three activity questions for osteoarthritis, back pain, and rheumatoid arthritis, which may be associated with PSP, were selected as predictive variables.

### 3.4 Personal data

**FIGURE 1:**
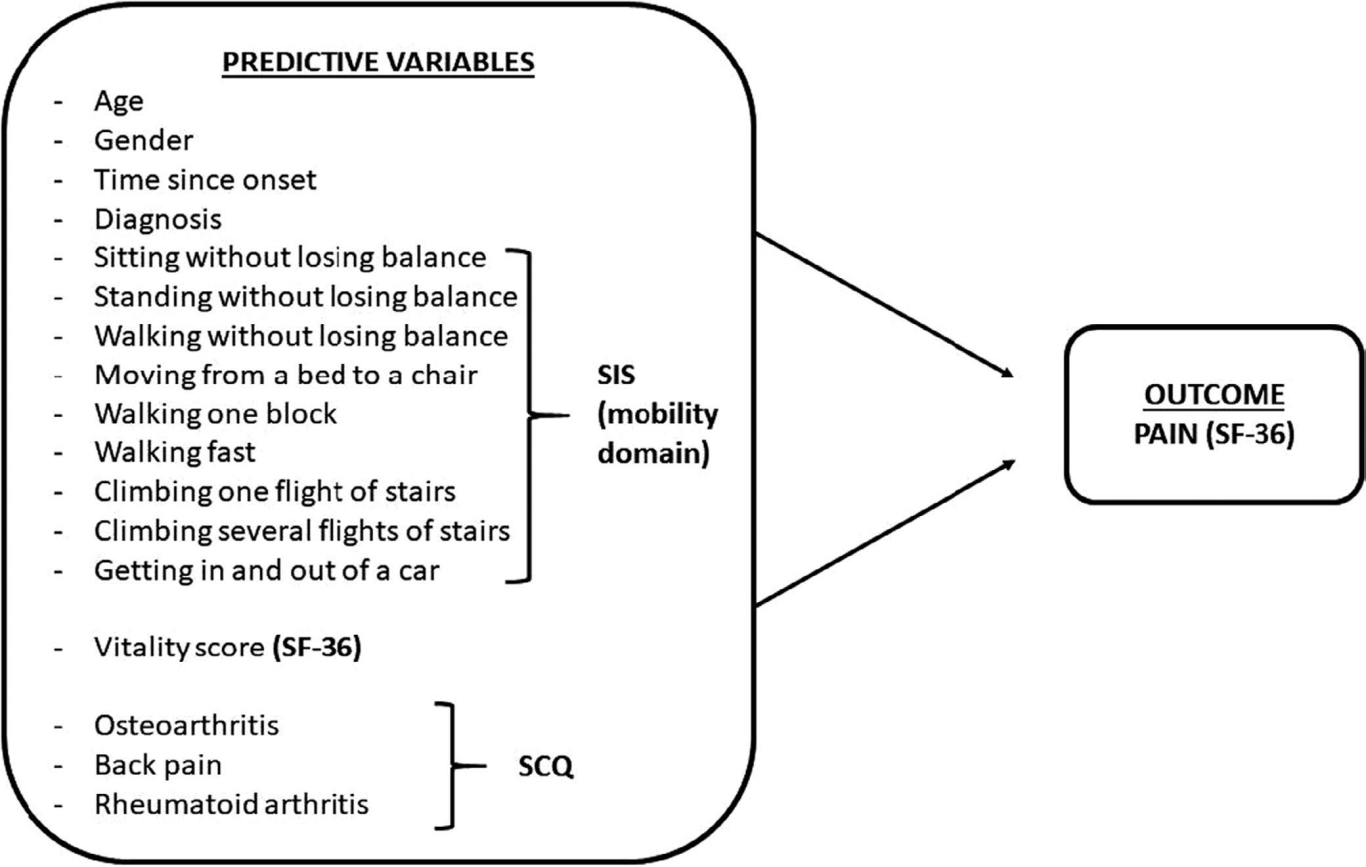
The possible predictors of Post‐stroke pain

Demographic and clinical information, such as age, gender, time since onset of stroke to interview date, and type of diagnosis, were other secondary predictive variables and collected from the patients’ files. The logistic regression model of this study, with 17 predictors and outcomes used in statistical analyses, is shown in Figure 1.

## 4 DATA ANALYSIS

SPSS 24.0 was used for statistical analyses. Descriptive statistics were applied for demographic data. To build a logistic regression model for explaining pain outcome, following steps were performed:

1. First, univariable binary logistic regression analyses were performed for each and every independent variable (17 variables—sitting without losing balance, standing without losing balance, walking without losing balance, moving from a bed to chair, walking one block, walking fast, climbing one flight of stairs, climbing several flights of stairs, getting in and out of a car, vitality score, patient age, patient gender, time since onset of stroke to interview date, diagnosis, osteoarthritis, back pain, and rheumatoid arthritis) and pain.
2. The independent variables with *p* <.25 after first step were included in a multivariable binary logistic regression.
3. A model with only significant independent variables to predict pain was left by excluding the least significant variables one at a time.
4. The variables which were excluded at step 1 were reinserted one by one in the model with the remaining variables from step 3. If a variable turned out to be significant (*p* <.05), it was decided to keep it in the model. (In this case, none of the variables turned to be significant *p* >.05).

The models built with the significant variables after step 1 and step 3 were compared with a Likelihood ratio test. It is decided that if the two models are significantly different (*p* >.05), the larger model which contains more variables would be chosen. If the models are not significantly different, the model with the least amount of explanatory variables would be chosen.

## 5 RESULTS

### 5.1 Patient characteristics

The patient characteristics are given in Table 1. The time after onset of stroke was almost 2 years (range 3 months to 13 years). Most of the study population was affected on the left side of the body (50.9%). 55.7% of the study population have reported pain, and the pain was more common in women. Also, the mean vitality score of women was lower numerically comparing man, which shown in Table 1. Our results also indicate that walking fast (73.0%), climbing several flights of stairs (63.0%), and walking without losing balance (53.3%) were the most reported items as being problematic by the participants of the study.

**TABLE 1:** Demographics, clinic characteristics, mobility difficulties, pain and vitality scores, and reported comorbidities of study population

Participants		Women	Men	All
		92 (43.4%)	120 (56.6%)	212 (100%)
Age, years	*Mean (SD)* *Median (range)*	67 (5) 71 (24–95)	66 (13) 65 (21–91)	66 (14) 66 (21–95)
Time since onset of stroke to interview date, months	*Median (range)*	25 (3–158)	22 (3–145)	22(3–158)
Diagnosis, *n* (%)	*Cerebral infarction* *Intracerebral hemorrhage* *Subarachnoid hemorrhage* *Stroke, not specified as hemorrhage or infarction*	50 (23.6) 6 (2.8) 10 (4.7) 26 (12.3)	50 (23.6) 18 (8.5) 9 (4.2) 43 (20.3)	100 (47.2) 24 (11.3) 19 (8.9) 69 (32.6)
Affected side of the body, *n* (%)	*Right* *Left* *Both* *Unknown*	37 (17.5) 43 (20.3) 4 (1.9) 8 (3.8)	40 (18.9) 65 (30.7) 8 (3.8) 7 (3.3)	77 (36.3) 108 (50.9) 12 (5.7) 15 (7.1)
Experienced difficulty in mobility domain of SIS, *n* (%)	*Walking fast* *Climbing several flights of stairs* *Walking without losing balance* *Climbing one flight of stairs* *Standing without losing balance* *Getting in and out of a car* *Walking one block* *Moving from a bed to a chair* *Sitting without losing balance*	67 (32.1) 67 (32.2) 59 (28.1) 54 (25.7) 47 (22.3) 42 (19.8) 48 (22.9) 23 (11.0) 11 (5.2)	86 (41.1) 64 (30.8) 66 (31.4) 58 (27.6) 58 (27.6) 61 (29.2) 45 (21.4) 40 (19.1) 15 (7.1)	153 (73.2) 131 (63.0) 125 (59.5) 112 (53.3) 105 (49.9) 103 (49.0) 93 (44.3) 63 (30.1) 26 (12,3)
Patient‐reported vitality score of SF−36	*Mean* *Median (min‐max)*	53.14 55 (5–100)	63.75 65 (15–100)	59.17 60 (5–100)
Having pain, *n* (%)	*Yes* *No*	25 (11.8) 67 (31.6)	69 (32.5) 51 (24.1)	94 (44.3) 118 (55.7)
SCQ—comorbodity‐related activity limitation—Osteoarthritis, *n* (%)	*Yes* *No*	10 (4.7) 82 (38.7)	11 (5.2) 109 (51.4)	21 (9.9) 191 (90.1)
SCQ—comorbodity‐related activity limitation—Back pain, *n* (%)	*Yes* *No*	17 (8.0) 75 (35.4)	19 (9.0) 101 (47.6)	36 (17.0) 186 (83.0)
SCQ—comorbodity‐related activity limitation—rheumatoid arthritis, *n* (%)	*Yes* *No*	4 (1.9) 88 (41.5)	3 (1.4) 117 (55.2)	7 (3.3) 205 (96.7)

### 5.2 Logistic regression analyses

The final regression model with only two predictor variables explained having pain after stroke significantly (*p* <.05). The results of this study indicated that stroke patients with problems in standing balance and those who have low vitality were 1.7 times more likely to have pain after stroke. Also, “standing without losing balance,” which was the item that 49% of the patients reported as problematic, was the only mobility item which was a significant predictor (*p* <.05). The results of the binary logistic regression analyses are given in Table 2, 3, 4.

**TABLE 2:** The results of the first step of binary logistic regression analyses

		B	Standard Error	Odds Ratio	p	Nagelkerke R Square
Age		0.002	0.011	1.002	0.884	0.000
Gender		0.684	0.298	0.505	0.022*	0.034
Time since onset		0.006	0.004	1.006	0.187	0.012
Diagnosis	Cerebral infarction	0.246	0.584	1.278	0.674	0.031
	Intracerebral hemorrhage	−0.951	0.487	0.386	0.051	0.031
	Subarachnoid hemorrhage	−0.784	1.439	0.457	0.586	0.031
	Stroke, not specified as hemorrhage or infarction	−0.209	0.336	0.812	0.534	0.031
Sitting without losing balance		−1.240	0.564	0.289	0.028	0.039
Standing without losing balance		−1.068	0.301	0.344	<0.001*	0.083
Walking without losing balance		−0.827	0.295	0.438	0.005*	0.051
Moving from a bed to a chair		−0.775	0.341	0.461	0.023*	0.039
Walking one block		−0.619	0.297	0.539	0.037	0.029
Walking fast		−0.916	0.320	0.400	0.004*	0.053
Climbing one flight of stairs		−0.501	0.290	0.606	0.084	0.019
Climbing several flight of stairs		−0.817	0.298	0.442	0.006*	0.049
Getting in and out of a car		−0.625	0.297	0.535	0.035	0.030
Vitality score (SF−36)		−0.033	0.007	0.968	<0.001*	0.140
Osteoarthritis (SCQ)		0.107	0.474	1.113	0.821	0.000
Back pain (SCQ)		−0.445	0.403	0.641	0.270	0.008
Rheumatoid arthritis (SCQ)		0.305	0.778	1.356	0.695	0.001

Note

SCQ: Self‐reported Comorbidity Questionnaire

SF‐36 Medical Outcomes Study Short Form 36

* *p* <.025 significant variables

**TABLE 3:** The results of the multivariable binary logistic regression analysis with remaining variables from step 1

	B	Standard Error	Odds Ratio	p	Nagelkerke R Square
Gender	−0.445	0.345	0.641	0.196	
Standing without losing balance	−0.733	0.416	0.480	0.078	
Walking without losing balance	0.172	0.444	1.187	0.699	
Moving from a bed to a chair	−0.154	0.435	0.857	0.723	
Walking fast	−0.653	0.398	0.520	0.101	
Climbing several flight of stairs	−0.027	0.427	0.974	0.950	
Vitality score (SF−36)	−0.24	0.008	0.976	0.003*	
Constant model	0.562	0.147	1.753	**<0.001**	0.210

Note

SF‐36 Medical Outcomes Study Short Form 36

* *p* <.025 significant variables

**TABLE 4:** The results of final model predicting post‐stroke pain

	B	Standard Error	Odds Ratio	p	Nagelkerke R Square
Standing without losing balance	−0.851	0.325	0.427	0.009*	
Vitality score (SF−36)	−0.027	0.008	0.973	<0.001*	
Constant model	0.585	0.146	1.795	<0.001*	0.182

Note

SF‐36 Medical Outcomes Study Short Form 36

* *p* <.05 significant predictors

## 6 DISCUSSION

The results of this study showed that restrictions in mobility and vitality are important factors associated with PSP. Having difficulty in standing without losing balance and low mental vitality were predictive factors of having PSP (*p* <.05).

Standing balance, which has a crucial importance for maintaining ambulatory and functional skills, is impaired after stroke due to sensory and motor deficits (Parsons et al., 2016). It is one of the strong predictors of independence in activities of daily living (Bohannon & Leary, 1995; Geurts et al., 2005). Some patients may stand independently first days after stroke onset, but a significant part of the stroke survivors report difficulties a few months later (Garland et al., 2003; Geurts et al., 2005). In our study, half of the participants reported difficulties in standing without losing balance even though the average time since onset is varied in subacute and chronic phases (Table 1). For those patients not feeling comfort and safe when standing, it can lead to inadequacy feeling and the use of compensatory strategies (Jones, 2017). The use of compensatory strategies can cause overuse and musculoskeletal pain. Therefore, compensations in stroke patients with different functional levels may cause PSP (Pain et al., 2015). This explains the relation between mobility restrictions and PSP in current study.

In our study, the time from stroke onset varied between subacute and chronic phases (3 months‐13 years). A study from Sommerfeld and Welmer suggested an association between PSP and mobility restrictions 3 months after stroke (Sommerfeld & Welmer, 2012). Additionally, mobility restrictions were found to be significant predictor for pain after 5 years of stroke (Westerlind et al., 2020). Contrast to these studies, time since onset was not a significant predictor for PSP in our study. In a study comparing the prevalence of different types of PSP in the acute, subacute, and chronic phases post‐stroke, patients in the subacute and chronic phases complained about pain more than those in the acute phase (Paolucci et al., 2015). Considering that all patients in our study were in the subacute or chronic phases, we can conclude that mobility restrictions in late phases of stroke are related to high pain levels, not the time since onset by itself.

Vitality was the other significant predictor factor in the model which explains the 36.5% of the PSP. It is known that such conditions like alcohol use and depression are some of the behavioral risk factors for PSP, and they also lead to low vitality (Delpont et al., 2018). Patients with low vitality have low energy and aliveness. It has been determined that depression and cognitive function disorders are important factors for recovery of different mobility tasks such as standing and climbing stairs in stroke patients (Geurts et al., 2005; Morone et al., 2018). From this point of view, low vitality may lead to pain indirectly by affecting mobility and directly by affecting the perceived pain level.

Previous studies that investigated gender influence on PSP have had different results. Although some of these studies showed that female sex is a significant predictor of PSP (Jonsson et al., 2006; O’Donnell et al., 2013) other studies detected no relationship between gender and PSP (Appelros, 2006; Klit et al., 2011; Lundstrom et al., 2009) similar to our study. Compared to men, women reported higher pain scores, but the gender was not a significant predictor of PSP. Women also had lower vitality scores, which is a significant predictor of PSP. These results may be because women feel more pain because they were more depressive than men not because they are women. It would be more explanatory if it would have been added psychological questionnaires to the study for patients to fill.

The location, cause, and type of pain were not investigated in this study, and some of a participant's overall pain score may not be directly stroke‐related, but related to, for example, physical inactivity or comorbidities. To eliminate the possible effect of painful comorbidities (e.g., osteoarthritis, back pain, and rheumatoid arthritis) on PSP, the levels of how much these comorbidities affect a participant's activities were included in the analyses. The proportion of people affected by comorbidities was between 3.3% (rheumatoid arthritis) and 17.0% (back pain; Table 1). According to the logistic regression analysis, no association exists between these comorbidities and PSP in the study population (*p* >.05). Thus, the additional possible causes of pain eliminated. In addition to this, it was not a predictor of whether the diagnosis was hemorrhage or ischemia (*p* >.05).

In the current study, the time since onset of stroke to the patient's interview date was not significantly associated with their overall pain score (*p* >.05). Previous studies evaluating the incidence of PSP and its subcategories have reported different results. The incidence of CPSP was demonstrated to increase with time at 1, 6, and 12 months after stroke (Andersen et al., 1995), but another study demonstrated that approximately one‐third of patients had PSP in the first months after stroke, with the proportion decreasing to 21% after 1 year (Jonsson et al., 2006). In contrast, we questioned only one pain score which answers the amount of pain. As the pain scores were calculated only once and the pain scores at different time points from the onset of stroke were unknown, the absence of an association between time since onset and PSP could be due to two things: The pain level remaining or decreasing with time.

The present study did not find that age is related to PSP, but some other studies (Klit et al., 2011; O’Donnell et al., 2013) have suggested younger people are more likely to have PSP. This results of our study may be due to the small number of young people in our study. In clinical practice, we do not see younger stroke patients as much as the older ones; thus, it is difficult to conclude that if they have more pain or not.

The current study has a few limitations. First, the types of PSP were not examined. This could give a better understanding by exposing the association between subcategories of PSP and mobility, as well as other factors.

Evaluating early and late pain scores for every patient would better explain the role of time since onset in stroke patients’ perception of pain. Although a sample with a broad age range is a strength of this study, dividing the sample into subgroups according to age and comparing them could give a better perspective of the relationship between age and PSP and other factors. Future studies could consider these steps to obtain more detailed results.

In conclusion, two predictor factors including poor standing balance and low vitality were important for stroke patients to have PSP and its effects on their life regardless of their age and gender. It may be advisable for rehabilitation professionals to consider the important role of restrictions in stair climbing and vitality in stroke patients to better understand the process of PSP.

## CONFLICTS OF INTEREST

The authors report no conflicts of interest.

## AUTHOR CONTRIBUTION

Sunnerhagen, KS. provided the data acquisition, concept, and design of the work. Atalan, P. analyzed the data with input from Berzina, G. Atalan, P., Berzina, G. and Sunnerhagen, KS. worked together on interpretation of the data. Atalan, P. drafted the manuscript. Berzina, G. and Sunnerhagen, KS. revised the article critically for important intellectual content. Everybody approved the submitted manuscript.

### PEER REVIEW

The peer review history for this article is available at https://publons.com/publon/10.1002/brb3.2092.

## Data Availability

Complete data cannot be made publicly available for ethical and legal reasons, according to the Swedish regulations (https://etikprovning.se/for‐forskare/ansvar/). Researchers can submit requests for data to the authors (contact: ks.sunnerhagen@neuro.gu.se ).
